# Another X-Ray Scare

**Published:** 1909-04-24

**Authors:** 


### Another X-Ray Scare.

There has recently appeared in the *Times* a letter signed by a well-known radiologist warning against the adoption of the report of the Sub-Committee of the Committee of the London County Council in favour of the treatment of ringworm by *x*-rays, on the ground that it is probable that the delicate cells of the growing brain of the child may be injuriously affected by even the short exposures used in this treatment, though the evidence of impairment of function may not become noticeable until development is complete. This suggestion, printed in the lay press and coming as it does from a medical man who describes himself as "an old *x*-ray worker," is likely not only to do much harm to a most valuable and well-tried method of treatment, but also to raise in the public mind a feeling of fear of the *x*-rays even worse than that produced by the bogey of *x*-ray cancer which has lately been constructed by the lay press. A little knowledge of the facts relating to the physical properties and the physiological action of the *x*-rays which have accumulated during the last ten years shows at once that such fears are groundless. The fact that during the past five or six years many thousand children have been subjected to this treatment in France and in this country, without the faintest suggestion of any mental disturbance, immediate or remote, ought to remove any doubt as to the harmlessness of this treatment. In the treatment of ringworm of the scalp it is the practice to give to any affected area a single dose of *x*-rays sufficient to destroy temporarily the hair-bulb, but not enough to damage the skin in any way. Such a dose is powerless to produce any changes in muscle, fibrous tissue, fat, or nervous structures. Even a considerably larger dose, sufficient to destroy the cells of the epidermis, will have no effect upon these deeper structures, because the cells of these tissues are very much less susceptible to the influence of the *x*-rays. In fact, this greater vulnerability of the skin forms a serious barrier to the therapeutic application of rays to the deeper structures. It will be seen, then, that so long as we apply to the scalp a dose which leaves the skin intact, we cannot damage the deeper structures. But in the application of *x*-rays to the scalp we have a further protection, in that the bones of the skull act as an obstacle to the passage of the rays. Even at the very thinnest parts of the skull-cap of a child not more than 30 per cent. of the rays go through, and, allowing for the fact that a large amount is also absorbed by the skin and fat of the scalp, not more than 20 per cent. of the dose reaches the cortex of the brain at any spot. These small doses received by the brain cannot be compared in strength and amount with those which led to dermatitis and subsequent troubles in early workers. These *x*-ray operators were exposed in many instances to enormous doses over short periods, or to constantly repeated small doses over longer periods, such as no one will ever be subjected to again, either in research or for therapeutic purposes. These doses led—in the first case within a few days, in the second case more gradually—to obvious destructive lesions, and any subsequent developments have been the result of these destructive lesions. There are no grounds whatever for supposing that a small amount of *x*-rays absorbed by a tissue at one sitting, and producing neither histological nor physiological changes, can have any long delayed result, injurious or otherwise.

To sum up, it may be said that, although a certain amount of *x*-rays does pass through the skull when a child's head is exposed, it is so small that, as the accumulated knowledge of years of experiment and practice tells us it is quite powerless for harm. To many who have only just become acquainted with this method of treatment for ringworm it may come as a surprise to learn that since 1903 some 500 cases have been cured annually at the St. Louis Hospital in Paris; that two or three London general hospitals for four or five years past have each turned out annually from 150 to 250 cures; that in several children's hospitals the treatment has been carried out on a smaller scale for an equal length of time; and that at the Metropolitan Asylums Board schools for ringworm over 1,000 cases have been cured by *x*-rays during the last two years.

